# Stories of taking part in Everyday Life Rehabilitation - A narrative inquiry of residents with serious mental illness and their recovery pathway

**DOI:** 10.3934/publichealth.2024062

**Published:** 2024-12-13

**Authors:** Rosaline Bezerra Aguiar, Maria Lindström

**Affiliations:** Department of Epidemiology and Global Health, Umeå University, Umeå, Sweden

**Keywords:** mental health, psychiatric disability, health equity, rehabilitation, recovery, activities, narrative, development and evaluation of complex interventions

## Abstract

**Context and purpose:**

Persons enduring serious mental illness (SMI) and living in supported housing facilities often receive inadequate care, which can negatively impact their health outcomes. To address these challenges, it is crucial to prioritize interventions that promote personal recovery and address the unique needs of this group. When developing effective, equitable, and relevant interventions, it is essential to consider the experiences of persons with an SMI. By incorporating their perspectives, we can enhance the understanding, and thereby, the design and implementation of activity- and recovery-oriented interventions that promote health, quality of life, and social connectedness in this vulnerable population. Thus, the aim of this study is to explore the stories of participants partaking in Everyday Life Rehabilitation and how they make sense of their engagements in everyday life activities and their recovery processes.

**Methods:**

Applying a narrative analysis, this study explores the stories of seven individuals with an SMI residing in Swedish supported housing facilities, participating in the Everyday Life Rehabilitation (ELR) program during six months, and how they retrospectively make meaning of their engagement in everyday life activities and recovery processes.

**Findings:**

The participants' stories about their rehabilitation and personal recovery pathways elucidate how the inherent power of the activity, as well as the support the participants received to get started and succeed, had a significant impact on their self-identity, confidence, motivation, mattering, life prospects, and vitality. The participants valued the transparent steps along the process, weekly meetings, the signals, beliefs, and feedback communicated throughout, and the persistent, adaptive, and yet supporting approach in their personal progress.

**Significance:**

This study underscores the need for interventions that prioritize meaningful activities and are sensitive to the complexity of the personal recovery process, especially in supported housing facilities. Future research should further explore effective strategies and mechanisms to promote personal recovery and to reduce the stigma associated with SMI.

## 1. Introduction

A person's mental health is essential at every stage of life, but it can alter throughout time depending on multiple and interacting social, psychological, biological, and environmental factors such as inequity, poverty, early adverse life experiences, and feelings of loneliness or isolation [1].

Mental health is acknowledged worldwide as an important aspect of public health. In September 2015, the United Nations (UN) recognized the impact of mental illness on the overall well-being, economic development, and social progress by including mental health as a priority in the Sustainable Development Goals (SDGs) [2]. This was a significant step, as it marked a global commitment to tackle mental health challenges involving all societal sectors, including governments, the private sector, community organizations, and the individuals themselves [3].

Accordingly, mental illness is a global public health problem with significant consequences for individuals and society. Serious mental illnesses (SMIs) result in enormous economic costs, with even more significant implications for economic growth than for the healthcare system [4],[5]. A significant fraction of years lived with disability (YLDs) [6] are caused by SMIs. People with an SMI often experience limitations such as poorer engagement in healthy activity patterns, including meaningful community involvement, low work capacity [7], self-care, sleep issues [8], stigma and discrimination [9], loneliness, isolation [10], and fear [11]. Consequently, individuals suffering from SMIs have their life expectancy reduced by 10 to 20 years due to a sedentary lifestyle and preventable physical diseases [12],[13], such as cancer, diabetes, and cardiovascular ailments [4].

Many SMIs can be effectively tackled, reducing individual suffering and economic costs [14]. People with SMIs need to be provided with comprehensive care that includes a range of health services and interventions. These should include pharmacological therapies, rehabilitation, and social support programs that enable personal recovery, meaningful activities, and social participation [15]–[17]. Nevertheless, effective treatments and rehabilitation are often hampered by underfunded health systems, a lack of qualified professionals, a lack of collaboration [18],[19], low status [20], stigma, and an inefficient use of health resources [4].

In recent decades, many mental health services and social support programs in most developed countries have adopted a personal recovery-oriented approach [17],[21]–[23]; moreover, some interventions, most of which focus on training staff, such as REFOCUS [24] and CARe [25], have personal recovery as the core focus. However, in Sweden, most organizations still need to implement a personal recovery paradigm [26]–[28].

“Personal recovery” is described as a deeply personal, unique process of changing one's attitudes, values, feelings, goals, and skills and/or roles [23], claiming that living a hopeful, fulfilling life, and participating in society, are still possible despite having a mental health condition [29],[30]. The acronym “CHIME” provides the five main contributing components in the process of personal recovery: connectedness, hope, identity, meaning, and empowerment [31]. A personal recovery approach recognizes the significance of engaging in meaningful occupations as a pathway to well-being and recovery [32],[33].

Individuals with an SMI living in supported housing facilities typically have a long-term and complex disability that requires ongoing support to manage their everyday life [34],[35]. However, living in supported housing facilities may further reduce self-determination, autonomy, motivation, and meaningful activities [36],[37]. In addition, collaboration between health and social care staff has been noted as a challenge [19],[26],[36], and the housing staff (HS) often presents poor levels of competence and lack belief in rehabilitation [19],[36],[38],[39].

People with SMIs often face stigmatization, discrimination, lack of access to resources [20], and limited opportunities [26]. A movement known as “occupational justice”, which is rooted in the broader social justice movement [40] and seeks to address issues of inequity and oppression in society [41], has pointed out these disparities. According to occupational justice, all individuals have a right to engage in diverse and meaningful occupations that promote their health and well-being [42]. As occupational beings, humans have a natural inclination to be active, to engage in activities that enable them to create meaning in their lives, to develop and express their identities, and to provide a sense of accomplishment and belonging within their communities.

However, vulnerable populations, including persons with SMIs that reside in supported housing facilities, are particularly susceptible to experiencing occupational deprivation. “Occupational deprivation” refers to a lack of opportunities or barriers that prevent individuals from engaging in meaningful activities [41]. It recognizes the impact of external forces—such as social, cultural, structural/organizational, economic, and political factors—on an individual's ability to participate in meaningful activities [41],[43]. The consequences of occupational deprivation may include poor health [15], capacity atrophy, and social exclusion [41].

Since people with SMIs who live in supported housing facilities often lead a sedentary life with a downward spiral of motivation, hope, meaning, and personal agency, and generally have low health literacy, they seldom actively seek rehabilitation themselves. In Sweden, basic healthcare, including rehabilitation, is mandatory for municipalities, which must offer basic healthcare to residents in sheltered or supported housing facilities [39],[44]. However, some municipalities lack rehabilitation, and in others, support for residents is insufficient [45].

Therefore, the Everyday Life Rehabilitation (ELR) program [36],[46]–[48] was developed to address the unique challenges of long-term housing facilities. ELR consists of web-based education for the HS and OTs, tools for collaboration, and methods targeted to the residents' personal recovery through enrichening activities and participation. ELR underlines occupational justice and equity regarding access to outreach, integrated, person-centered, long-term, and timed activity-based and recovery-oriented rehabilitation, promoting a meaningful everyday life, personal recovery, and an increased quality of life [48].

In order to develop and refine relevant interventions, it is important to include the perspectives of the intended participants. Drawing on the experiences of persons with SMIs who have recently participated in the ELR, this study aims to give them a voice. Interpreting and illuminating their narratives permits their voices to be heard and recognized, empowering them to be active agents and promoting their inclusion in discussions related to their recovery processes [49]. Narrative analysis is a valuable method to understand the sense-making of intricate ways in which the recovery process unfolds through the activities and experiences of everyday life [50]. Using this approach, the researcher can identify the key events and meaning-making within each participant's story, which can help further clarify the meanings and interpretations they attach to their experiences [51].

Narrative analysis [52] offers a means to explore and illuminate the stories of individuals with SMIs living in supported housing. It allows them to express their experiences, perspectives, and insights [52], which are often marginalized or overlooked [20]. Narrative analysis enables a deeper understanding of the complex interplay between activities, personal experiences, and the broader context in which recovery occurs [50],[33].

Adopting a health equity perspective, this study highlights the need for interventions that address occupational deprivation and provide individuals with SMIs living in supported housing facilities equitable opportunities to engage in meaningful activities [53]. Consequently, this study seeks to contribute to the body of knowledge that informs the development of more person-centered, activity- and recovery-oriented approaches that address the unique needs and preferences of individuals with SMIs residing in supported housing facilities, promoting a healthy and meaningful everyday life, personal recovery, social connectedness, and quality of life.

The aim of this study is to explore the stories of participants partaking in ELR and how they make sense of their engagement in everyday life activities and their recovery process.

## 2. Methods

This study applied a narrative analysis [52] based on the participant's stories. The narrative analysis approach was considered suitable as a means for the participants to reflect upon, make sense of, and communicate the meanings they attributed to participating in ELR and meaningful activities in relation to their recovery process.

Through a narrative analysis, the researchers collect detailed descriptions of the events, actions, and experiences as the primary source of data [52]. The basis of the narrative inquiry is the idea that individuals create meaning by narrating their stories. Additionally, the collaborative aspect of narrative research allows for interactions between the researcher and participants, enabling them to co-create knowledge, as meaning-making occurs in a collaborative process [54].

### 2.1. Overall context

Complex mental health-supported housing accommodation services in Sweden are based on a needs assessment and are mainly regulated by the “Social Services Act” [55] to ensure a reasonable standard of living, or the “Act Concerning Support and Service for People with Certain Functional Impairments” [56] to ensure good living conditions for people with extensive and persistent disabilities, including integrated basic healthcare and rehabilitation, according to the Healthcare Act [44].

In Sweden, there is a considerable variation in the praxis of supported accommodations, particularly regarding offers of integrated rehabilitation. A movement toward a more recovery-oriented direction and toward individuality has been noted in some municipalities in Sweden [57], though this has still not been implemented at large. Some municipalities have no rehabilitation services at all, while some offer only one-off interventions, usually in the form of prescribing aids; only a few offer integrated, long-term rehabilitation in collaboration between rehab staff and HS. Additionally, collaborations between these roles and responsibilities have been pointed out as a challenge, and the competence level among the HS is generally low [39].

Therefore, the ELR model was offered to and implemented within municipalities in northern and middle Sweden that agreed to take part in a Randomized Control Trial (RCT) project [45], including a web-based educational package for the rehab- and housing staff, which examined the effects of ELR compared to “treatment as usual” from the perspective of residents' personal recovery and quality of life. The full project was pre-registered at ClinicalTrials.gov, NCT05056415. Parallel to the RCT, the research team also wanted to understand how the participants made sense of and described the rehabilitation they had just undergone with the ELR, forming this narrative inquiry study.

### 2.2. Intervention—The Everyday Life Rehabilitation (ELR) intervention

Given the health inequity and activity deprivation of the target group and the scarcity of collaborative and integrated re/habilitative methods in the supported housing accommodation context, the ELR was designed and developed [36],[45]–[48] based on the best evidence for different mediating components and experiences from user-, praxis-, and stakeholder representatives to meet these challenges and legislative demands, and to improve and transform the rehabilitation efforts towards more person-centered, motivational, activity-, and recovery-oriented efforts.

**Figure 1.**
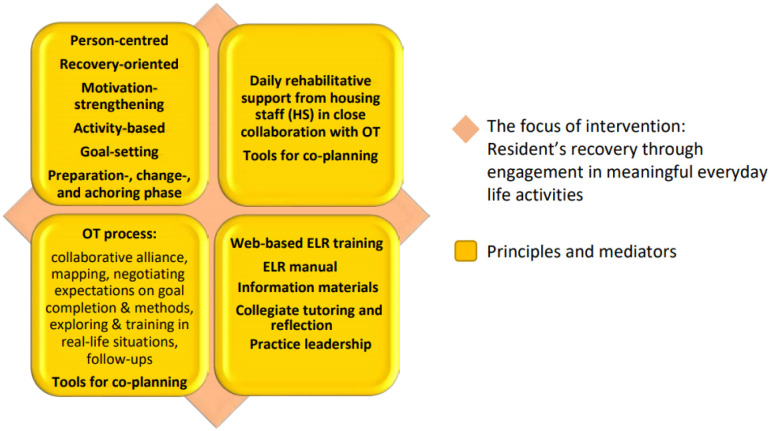
The Everyday Life Rehabilitation (ELR) intervention.

The focus of the intervention is to promote personal recovery through engagement in personally meaningful and enriching activities chosen by each participant and supported by occupational therapists (OTs) and the housing staff (HS) [47]. ELR is a complex intervention that targets complex needs in a complex context, including several interventional components and actors: residents, OTs, HS, and managers (Figure 1).

ELR was delivered through integrated rehabilitation based on “outreach” efforts directly to residents, applying a preparation-, change-, and anchoring-phase, described in an invitation brochure as a personalized opportunity of change towards increased quality of life, enrichening activities, and personal recovery.

ELR was provided by weekly encounters with OTs, building a personally designed rehabilitation plan with a duration of six months, and next, in line with the plan, exploring and training in real life activities based on the participant choice to gradually reach the personal goal. In parallel, and in close collaboration, daily support was provided by the HS. The approaches of the professionals promote hope, self-discovery, and shared decision-making shaped in partnership with the participant/resident (see Figure 1).

### 2.3. Recruitment, participants, settings, and data collection

The narrative analysis seeks depth rather than breadth of experience. Therefore, a smaller number of participants rather than a larger sample size is recommended to deeply understand the subjective reality of the participants. This study used a purposive sample of individuals with SMIs living in supported housing facilities who had participated in ELR.

The participants were recruited from the municipalities involved in the RCT project during waves 1 and 2, via an information- and consent-form distributed by either research assistants or occupational therapists already involved in ELR to recruit the participants. A total of seven participants from three different municipalities agreed to take part in this narrative study and shared their experiences of engaging in ELR (Table 1). The participants lived in two different types of housing contexts: type 1 and a hybrid of type 1 with elements of type 4, according to the “Simple Taxonomy for Supported Accommodation” (STAX-SA), developed by McPherson and colleagues [57],[58].

**Table 1.** Participant characteristics.

Person	Sex	Age	Type of housing	Municipality
1	Man	39	^1^Type 1	Medium size
2	Woman	47	^1^Type 1	Medium size
3	Woman	55	^2^Hybrid (1 and 4)	Small size
4	Man	40	^2^Hybrid (1 and 4)	Small size
5	Man	58	^2^Hybrid (1 and 4)	Small size
6	Man	52	^1^Type 1	Medium size
7	Woman	38	^2^Hybrid (1 and 4)	Small size

Note: ^1^Type 1 residential care homes with staff on-site 24 hours, organized as congregate settings for several tenants (2–14) with complex needs. ^2^The service units here framed as hybrids between type 1 and 4, resembled type 1 in most respects, but deviated in the domain of physical setting; individual instead of congregate, and a variation of staff location; in some sense outreach service because staff were located in another building. None of the accommodation service types had any emphasis on the “Move-On” approach.

Data were collected through conversational interviews to elicit the individuals' subjective experiences in a narrative form. The interview was comprised of open-ended questions asked as icebreakers for contextual conversations. The participants were prompted to share their experiences of being involved in ELR, to give examples of how the process evolved, and the meaning they ascribed to the personal activity goals they focused on.

The last author (ML) conducted three of the interviews in 2022, with two interviews taking place in person at the residents' homes and one conducted via Zoom and recorded via a separate device, based on their preferences. The first author (RBA) conducted four of the interviews over the phone during spring 2023. One participant was interviewed twice to collect additional information for specific questions. The interviews were conducted in Swedish, lasted between 10 and 90 minutes, and were audio recorded.

### 2.4. Analysis

A narrative analysis was applied to focus on the stories shared by the participants and how the stories were told in relation to the context. During the analysis process, the authors held numerous meetings to discuss analytical steps, preliminary findings, and possible interpretations.

The interviews were first transcribed verbatim and read several times to ensure a thorough understanding of the stories. Subsequently, the authors identified and discussed significant events, and then reviewed the transcripts again, identifying potential plots and subplots relevant to the meaning-making and research aim. The emerging plots were connected to gain a comprehensive understanding of the story and to organize them into “an intelligible whole” [54]. The storylines were analyzed to reveal how the story was constructed and conveyed meaning.

As part of the analysis, different theoretical illuminations were applied to find the best way of adding a further understanding to the participant's stories [52]. Several possible interpretations were tried and negotiated, resulting in the final interpretation that best illuminated and agreed with the raw data and increased understanding of the participants' experiences and stories. It was evident that many aspects of the participants' stories shared similarities, while some differences stood out. Three participants spoke in depth about how the chosen activity influenced their personal growth, while four participants mainly spoke about and attributed the merits of the methods and the support they received as the most important aspects of their change process. This is why three participants' stories were reconstructed as coherent stories on the power of meaningful activities, while all seven participant stories contributed to the storylines about how personalized support enabled personal progress.

The process of analyzing and reconstructing the narratives was iterative and ongoing, following the principles of a hermeneutic circle [54], with the researchers elaborating and refining the stories as they further analyzed the data. This process aimed to develop a comprehensive and nuanced understanding of the experiences and perspectives of the individuals involved in the study [51],[52] and reconstructed them into illuminative stories to best increase the understanding.

### 2.5. Ethical considerations

This study was approved by the Swedish Ethics Authority, Dnr 2020–06220. The participants were informed about the purpose and procedure of the research, and written consent was obtained from all informants. Additionally, they were informed that their participation was voluntary and that they had the right to withdraw from the study at any time. All names were changed to ensure participant confidentiality, and any identifiable information was removed from the data sources.

## 3. Findings and theoretical illumination

The findings are presented in three parts: first, in three personal recovery processes, where the inherent power of the activity had a significant impact on the participants' self-identity, confidence, motivation, and vitality; second, in participant storylines, illuminating how the personalized support enabled them to take an active part during their process of personal progress; and finally, we present the theoretical underpinnings of our analysis. These narratives, reconstructed here as coherent stories, are chosen because they are representative of the participants and illustrate the potentially transformative impact of participating in ELR. Verbatim quotations are written in italics and within brackets, and the personal names are changed.

### 3.1. Three recovery processes where the inherent power of the activity had a significant impact

#### 3.1.1. The power of nature: a transformative experience of self-discovery and reconnection with life

Anne is an inspiring and creative woman in her 40s with a powerful story about her journey of rediscovering herself, her self-worth, and finding purpose in life. Before partaking in the ELR program, Anne described herself as hopeless and as having no self-worth, identity, or motivation. She was facing a difficult period in her life, feeling lost without a clear sense of direction, which made everything seem dull and colorless. It was as if life had no meaning for her.

*My life had been slipping away over the years. I did nothing, was not my own person, and had no value. I was wiped out as a human being, completely passive, without any driving force*.

Nevertheless, when Anne read about ELR, she felt a small spark of hope, and she became curious about it. Some words in the information sheet stuck with her, and she felt invited to explore it further. This small amount of hope and curiosity ultimately led her to participate in the ELR program and begin her journey toward rediscovering her self-identity and connection to life.

*It was a little hope and... a curiosity that was awakened. There were a few words I got stuck on in the information leaflet, the invitation about this. Yes, it was to enrich everyday life. I felt that was what I would need before it was too late. I was about to cease being my own person, give up, let myself flow away... it sounded like all the power did not have to come from me... that I could get a little more power by enriching my everyday life*.

At the beginning of the rehabilitation, Anne was unsure about what activity to invest in and whether she would be able to carry out an activity due to her current condition. However, reconnecting with herself and exploring her interests and abilities allowed her to find activities that suited her conditions and encouraged her. Ultimately, Anne chose to experience nature, and the OT suggested that she could combine it with creative writing based on her experiences.

*I chose to experience nature! I have always appreciated nature but forgot the power of it in the last 20 years of my life. I actually had another option too; I thought of something creative because earlier in my life, when I was young, I have also been doing creative things, but not in recent years either... but I still chose to experience nature first-hand... felt that it could give me the most and enrich me the most...*

According to Anne, the most challenging part was finding the energy and will to do something for herself, as she had lost all the energy in her body and had forgotten the capacity she held within herself. Even though it was a major step for her to get into nature, she had a great experience during her first outing, which motivated her to continue exploring and engaging in outdoor activities.

*Yes, it was a strong experience; I wanted more. I want to continue. I felt it gave me something fresh and soothing; it filled me with a lust for life and calmness. I felt balanced, fulfilled, uplifted*.

During her rehabilitation, she started with small progressive steps supported by the OT, such as going to a nearby forest path with a blanket, finding a clearing, and sitting down to look at the forest, breathe, and take nature in. According to Anne, the experience was simple and effective and was enriched by the many impressions that nature gave her. From there, she continued to explore nature's power through various activities, such as going for walks, collecting materials, and creative writing based on her nature experiences.

For Anne, experiencing nature was a turning point for her. She explains how she found her way back to nature and discovered its healing power, which gave her energy, balance, and belonging, and helped her to cope with her challenges. Moreover, she mentions that she discovered essential things, such as the nuances, colors, smells, and the beauty of nature, which helped her regain positive feelings such as hope, believing in herself, contentment, and self-worth as a human being. This helped her overcome her initial doubts and uncertainties and gradually build her confidence and sense of self-efficacy.

*Hope for life and some meaning in that life... support to rediscover the healing power of nature. I would say I have gone from a life of vagueness and slow disintegration of myself, with anxiety pounding on without being able to do anything about it... to... to filling life with a little more meaning, some positive feelings, to seek impressions from other environments, discover nuances, discover feelings, discover what I want...discover myself, let myself take part in life, nature, impressions..*.

This indicates that the rehabilitation helped Anne gain a new perspective on herself and her life. She recounts feeling more positive and having a sense of meaning in everyday life, representing a shift in her overall attitude toward life. Rediscovering the healing power of nature played a role in helping Anne view herself in a new and positive way. Self-discovery was a gradual process that involved exploring new environments, discovering nuances, and allowing herself to be a part of life. This newfound connection to nature allowed her to tap into her inner strength, confidence, and creativity, which she was not previously aware of.

*... did I say I have written a couple of poems too? It started with single words but turned into lines, short lines that I can look back on as a turning point when I let nature make room in my life again*.

Anne engaged in meaningful activities that allowed her to establish a connection with herself and the world around her. This enabled her to discover herself, find new passions and a sense of purpose, and awake to a deeper appreciation for life. Anne claims that the experience in nature was transformative and had a significant impact on her overall well-being. According to Anne, the HS will provide ongoing support to help her maintain her engagement in activities in nature after the rehabilitation period, even during the winter.

#### 3.1.2. Transformed through voluntary work: personal growth, value, and a sense of accomplishment

Nils is a man in his 30s who has always wanted to make a difference in other people's lives, but he says he was unable to act on it until he received the support, guidance, and structure provided by an OT. Nils chose to participate in ELR because he was attracted to the personally-oriented rehabilitation and the opportunity to enhance his life through meaningful activities, as described in the leaflet he read about ELR.

*I read the leaflet and thought, why not? I will not become a serf. I can stop if it does not feel good (laughs)... then I thought... that it might still enrich my life a little more, as it said in the leaflet. That was probably what caught my eye, a personally oriented rehabilitation. Why not? That is something out of the ordinary*.

Despite Nils' previous sedentary lifestyle and low energy levels, he tells he was willing to take on his first challenge: choosing the activity he would invest effort in during the ELR program. He describes that he had several ideas in mind for activities, and with the OTs guidance and questions about his values, interests, and aspirations, he could narrow down his options. Eventually, he chose an area he had been thinking about for a long time—helping older people with outdoor tasks.

*Yes, it felt meaningful and important to be able to help someone else. I do not mean much to anyone else, but I felt that I wanted to do it, that I wanted to be able to help someone else*.

Nils explains how it was important for him to choose an activity that was not just temporary or superficial, but rather something real, challenging, and meaningful that would benefit someone else. The first person he chose to help was a relative on his mother's side, who had difficulty walking and needed help with outdoor chores. Consequently, his second challenge was, together with the OT, to create a plan to help him achieve this goal. The plan was designed to prevent him from overexerting himself and to help him establish a routine. Nils remarks that the plan was based on gradual steps to help him succeed and to avoid failure, which was important to him.

*... I was given a plan so that I would take it step by step, not go out too hard at the beginning, so to speak... it was probably good not to make too big of promises either. The Occupational therapist is the one who made the plan concrete, and she accompanied me during the first few months until I had established a routine of how I would do it*.

During rehabilitation, Nils recounts how he dedicated his time and effort to various physical activities, such as shoveling snow, chopping ice, sanding, cutting grass, and raking, to help his elderly relative. He found these activities to be physically challenging, but also gave him a sense of satisfaction of having done something real. He was motivated by the challenge of the tasks and his desire to help someone in need, which allowed him to overcome the difficulties he encountered.

*Very significant! I feel that I am helpful. It gives me a good feeling in my body when I have exerted myself physically; I feel tired but satisfied. I feel that I can make a difference in someone else's life. It feels incredibly meaningful... and I have received nice thanks from Joakim; he is incredibly grateful for what I do..*.

Nils acknowledges that the physical challenges and fear of failure were not the only obstacles he faced during the rehabilitation period. He also experienced some resistance and apprehension when faced with the tasks he had to complete. Nils recounts there was one instance when he did not feel well and could not complete a task as planned. Despite occasional difficulties, Nils successfully carried out the activities according to the plan and adapted to the weather conditions. For instance, during mid-January, there was plenty of snow shoveling to be done, and although Nils felt he could have done more, he expressed satisfaction with his commitment to the plan.

*... sometimes I have felt resistance or some kind of apprehension about whether I will be able to do it, but it has still gone well... there was probably one time when I was not feeling well that I could not do what we had said and planned for... but all other times I have done the work according to plan and according to the forces of the weather [laughs]*.

By successfully achieving his initial goal, Nils' self-confidence increased, making him feel more proactive, self-motivated, and empowered. Nils mentions that he started going to Joakim's place more on his own, doing chores, and even calling his relative to ask whether he wanted anything done in particular. In a way, he exceeded his own expectations by pushing himself further than he had initially planned. As Nils continued his volunteer work, he began to receive positive feedback and recognition. This validation of his voluntary work helped reinforce his motivation and value as a person.

*... Towards the end, it has probably also been the case that we had not done as much practical work together but talked more before and after about how it went and how it felt. I have been told I have become like a farmhand and gardener—it felt good*.

*My mother is also proud that I have been able to help Joakim so well. She says that it is joyful to watch and that she also feels that I have felt good doing it*.

Nils expresses satisfaction about his progress during ELR and is motivated to keep improving with the support of the HS and the OT after completing the rehabilitation period. According to Nils, engaging in voluntary work has been a transformative experience for him. He believes that extending a helping hand to others has brought him a sense of purpose and significance. He has developed confidence in his ability to take on new challenges, which can help him to succeed in other areas of his everyday life.

*I have simply been helpful while I have lifted myself up, or rather, these outdoor pursuits have lifted me up and given me value. I have felt it has been something substantial I have done. I have taken it on, got sweaty, had muscle pains from exercise, and it has given me a nice feeling inside. I have grown as a person; partly that I have taken hold of something, made a change in my life, with support and a little pushing, of course, but still, that I have managed it, and partly that I have regained faith in my ability to get to work. I have felt good about it, and also that I have made a difference in an old person's life, a relative's life... it has felt valuable in many ways*.

Nils' experience with voluntary work during his engagement in ELR demonstrates the transformative power of meaningful activities. By choosing an activity that was aligned with his values, interests, and aspirations, Nils was able to find a sense of accomplishment and purpose in life.

#### 3.1.3. Connected, confident, and motivated: overcoming fear and regaining a zest for life

Gustav describes himself as a person who has been struggling with low self-esteem, a lack of confidence, hopelessness, and had lost faith in himself for a long time. He depicts a very powerful story illustrating how ELR has helped him rekindle his motivation, overcome his fears, regain control over his life, find a sense of belonging, and rediscover his enthusiasm for life.

*I think it has been really... For me, it has actually been life. Well, I would not say that I have considered suicide, but I was so depressed with how my life was before ELR... that I just went through the days. I usually say that I was alive but not living; I was just... I sat and watched a lot of TV and did not get anything done. And ELR has really helped me get back my lust for life*.

Gustav describes that he was unmotivated to engage in productive activities and had a pessimistic outlook on life. Nevertheless, his participation in the ELR had a significant impact on him, allowing him to recognize his strengths and qualities and appreciate the small positive things in his life that he was previously unaware of.

According to Gustav, the OT helped him set personal goals and took conscious, planned, and supervised steps to achieve them. Gustav was motivated to engage in meaningful activities such as slalom, and social contacts, which were big steps for him. In order to reach those goals, he was encouraged to start practicing yoga, work out at a gym, take short walks, and gradually increase his physical capacity alongside a physiotherapist (PT). Additionally, the OT encouraged him to take even more significant steps, such as attending the opera, which he describes as a revolutionary experience. He loves music and had never been to the opera before.

*I am a film and music geek, so I enjoy all types of music, and I usually joke and say that if someone has a favourite artist or group, I have probably listened to them. I had never been to the opera, even though I enjoy it. Then last fall, the big leap I took was to go to the opera. So, it was really... It was a huge... what an experience [laughs]*.

By achieving these goals, Gustav was able to overcome any doubts he had about coping with his situation. This experience provided him with a sense of accomplishment and self-belief, and boosted his self-efficacy and motivation, which encouraged him to set more ambitious goals and work towards them in the future. Gustav gained a new perspective on life and began to break down the obstacles that had previously hindered him from having a functional daily life.

*I love slalom and have not skied in ten years. And ELR has also helped me to make contact and get help to get started... I do not even cycle nowadays. And then with ELR, it is like... It gave me such self-confidence that I have got started with it*.

As Gustav started to explore his potential, desires, and interests, he became more confident and motivated to achieve much more in life. One of the ways he was encouraged to do this was by participating in the local pilot leisure project, which provided opportunities for social contacts and personal growth. He recalls how it was so much fun to have the freedom to wish for exactly what he wanted to do. Gustav yearned for a companion who shared his passion for movies and believed that there were others who also felt alone and wished for someone to go out with or simply share a cup of coffee. They started meeting up, grilling sausages, and hanging out.

*It was so fun. You could wish for whatever you wanted. So, they really tried to put this together like ...me, people who love movies. I would like to have a movie buddy to go with. I thought that there were more people like me who are alone, sitting in an apartment, and love movies and have no one to go with, or just to go and have a coffee*.

The leisure group was more than just a pastime for Gustav, as it brought him valuable connections, motivation, companionship, and friendships. He speaks highly of the group, which has become a crucial part of his social life and provides him with a sense of support. This helped him overcome isolation, loneliness, build confidence, and take control of his social life.

*And then the great thing about leisure time is that they have this idea that... So, they call and... well, we have Mondays and Fridays when we get together. We grill sausages and just hang out as a group. And if someone does not show up, they call to check that they are not stuck at home*.

Gustav speaks very positively about ELR, describing it as one of the most positive experiences of his life. He appreciates the tools he has gained to make positive changes and to live a more meaningful life. He is filled with gratitude for having participated in the ELR program and considers it to be a truly life-changing experience.

*... that finally, this tool has come, which helps one move forward in life and find something that makes life meaningful. I just think it has been so positive. So, when I am going to rate it, I tell OT that... it is mostly tens. And then she told me that in September, during the first interview, I had a maximum of four out of ten regarding my views on life and such. And now, I think I had a minimum of seven, or six or seven. I do not have a job, so that is what brings my rating down. But otherwise, I think it has been absolutely the best thing that... after my nieces, this is the best thing that has happened to me in life*.

With the end of ELR, Gustav admits he is afraid of slipping back into his old ways. Moreover, he suggests that the leisure group should organize more in-person activities, such as watching movies or visiting the bathhouse, to facilitate socialization and connection among members. Additionally, Gustav conveys that he still has difficulties leaving his home and suggests that he would have benefited from tips and support to improve his social skills, which he believes would enhance the overall experience of being part of the ELR group.

*That I get stuck... at least I do, and I still have some difficulty with this social aspect and saying... Even though I can sometimes understand that a person has the same interests, I have such a hard time with it, to... yes, to get help with... and get tips on how to learn this thing about making social contacts with people*.

However, Gustav recognizes the great progress he has made through the ELR program and with the help of his OT and PT. He is committed to continuing with this positive mindset and making gradual stages toward a better and richer life. Gustav claims he is determined to take advantage of opportunities and say yes to social outings, such as going to the movies with a friend he had not seen in a long time.

*... Because I have talked to OT about and I have said... I have been afraid that when this ends, I will slip back into my old... well, my old life, when I just... well... where I do not care about doing anything during the day. But I feel now I have received such good help from ELR and a good foundation in my own self-esteem and self-confidence, so I will continue to think that way. I will continue to take advantage of these small steps and these chances you get in everyday life; for example, hanging out with a friend*.

According to Gustav, he has experienced a notable improvement in his self-esteem and confidence, which has enabled him to rediscover his passion for life. He acknowledges that he had lost faith in himself and his ability to contribute to the world. However, through the support and guidance provided by the ELR program, Gustav has been able to rediscover his self-worth and recognize his positive qualities.

*Well, it is about finding self-esteem and self-confidence within myself again because I had lost it. I lost the trust, comfort in myself, trust in myself, that I also have something to contribute. That I am also a person who... well, but has... yeah, has good sides and qualities*.

He also puts into words a special event where he discovered a new feeling of pride, which at first, he did not recognize:

*When I walked out of there, I felt sick. I seriously thought I was sick to my stomach. And then I thought to myself, no, what I am feeling is that I'm proud of myself and satisfied... And it has been so long since I felt proud of myself, so I thought I was sick when I felt that feeling [laughs]. I have become good at patting myself on the back, thanks to ELR. But it's been really... incredible... I will remember this for the rest of my life, this help, this half-year that I got to be part of this project with ELR... I will remember it. I will do that*.

### 3.2. Storylines about support in exploring and progressing

Parts of participants' stories center mostly about what they appreciated and how they recognized significant support and progress along their change process. They weave together their own experience in the form of examples of how they got started and what kind of changes the program brought in their everyday lives, with how they actively experienced their own rehabilitation processes and the support they received from OTs, the HS, and in some cases, the PTs.

Three prominent storylines were crystallized in the material, on how the support enabled them to take an active part during their process of personal progress, as described below.

#### 3.2.1. Awakened motivation for change and personal progress

Increased hope of personal development, hope of positive change, and encountering intrinsic motivation for personal progress were expressed as a process of awakening.

Lisa's account is really strong when she recalled the following: *“It has attracted one's own inner motivation, belief in possible changes, and trust in life, one's value as a person, insight into what gives meaning and matters, and to dare to realize how to influence one's everyday life.”*

Gustav describes how astonished he was by acquiring motivation and experiencing so many changes in such a short period of life: *“I am willing to share all this with media or anyone interested, I don't care about secrecy, I want to tell everyone about my journey and the help I received from the occupational therapist and physical therapist together... all the new achievements I have conquered and activities I have started... I have found motivation, friends, I have conquered health issues, I have started with many new activities and social associations, I have fulfilled dreams...”*

The approach seems to have attracted inner motivation, realizing possibilities and trust in life, potential, one's value as a person, meaning, and being able to positively influence one's everyday situation. The participants formulate that the OT, the PT, and the HS combined have been encouraging; to dare to say yes, dare to do something new, dare to invest in oneself, with the right to drop out if it does not feel good. The participants point out that it has felt good that someone believes in you, to get a feeling that something positive can happen, and to believe in oneself and the opportunities in everyday life.

#### 3.2.2. Weekly meeting with occupational therapist; an undisturbed moment clarifying me and my own will

Several participants expressed that they particularly appreciated the weekly meeting with the OT: an undisturbed moment that is only one's own. They state it has given confirmation and encouragement to be listened to, to be supported to think about values, who one is, what one wants, how one wants it, what one desires from everyday life, and eventually a discovery about how one can positively influence it by doing things that give meaning and impact to one's everyday life.

Kristina elaborated on her telling and affirmed the importance of personal time: *“I have appreciated the time alone every week with my occupational therapist, she has been so good at listening and attracting me to my own will, daring to tell and do more, and discovering things about myself.”* Lisa voiced: *“and with that help, I can find out what I really value in myself and wishes of my life.”* Erik expressed: *“Receiving praise and very concrete support has given me the courage to dare a little more all the time and grow in value as a person, who I am, daring to express my will, and discover opportunities around the corner.”*

The amplification of personal interests, values, challenges, wishes, identity, and self-worth appears to have helped the participants clarify who they are, what they want, and how to express their own will, ranking, and choice of activity in which to develop and flourish.

#### 3.2.3. Clear process and communication—tracking personal progress and adjustments

When the participants spoke about their experiences participating in the ELR, they used phrases such as Lisa: *“it was very well laid out, which altogether stimulated a drive for personal development”;* and Nils: *“everything from the invitation, to the choice of activity and goal-direction... the support, and the different steps and phases communicated verbally and in the personal plan”*.

The participants described that it felt like a well-thought-out method and that they appreciated being told about the various steps in the process, how long it might take, and how it could be done, and moreover, how the personally designed rehabilitation plan made the process feel extra resourceful and effectual. They appreciated that the goal was like a guiding light in terms of what you were aiming for together with structured strategies; however, it could have been even more well-organized in terms of who was supposed to do what, according to one account. Furthermore, they appreciated the orientation and tone of the worksheets, even though some words were difficult, but also welcomed the messages and treatment from the staff. A transparent process and validating communication assisted their personal progress and personal agency.

Peter, who previously got stressed by his organized social- and work-training, and whose goal in the ELR-period was to work in strategies for taking breaks during the day, really appreciated the clear strategies that the OT helped him with: *“It has been great...We alternate the practical with sitting down and talking through, and I feel confirmed and strengthened, that I have a right to take breaks, and clear strategies about what I can do, and when, to take helpful breaks, so that I can endure more during the day.”*

Even when the participants faltered in their motivation, felt unwell, or appointments were cancelled, the persistence from both the rehab staff and the HS was maintained, according to the participants, which helped them get back on track. Flexibility, appropriate pushing, and finding alternative ways or adaptations in the rehab plan were expressed as extra important during the more difficult periods. Kristina acknowledged the following: *“The extra pushing when needed, and adjustments suggested along the way, helped one to continue to believe in being able to succeed in achieving one's goal, despite certain setbacks at times.”* Lisa added: *“The extraordinary engagement, tracking personal progress, and feedback communicated recurrently, were really effective.”*

All participants emphasized the importance of personal plans and recurrent feedback, together with achieving the goals set during rehabilitation. Most participants achieved their goals and expressed a sense of pride and joy, except for one who was not entirely satisfied with her quality of goal achievement. They stressed the significance of beginning with gradual steps and progressing towards more difficult challenges, gaining confidence and motivation along the way. Gustav claims: *“ELR has done an amazing job. It has really addressed what a person needs to get support and help to get started and... well, to feel that I am actually satisfied with the work I did on myself. Because that is when I, that is when I am really satisfied with the job that I have received help with, to find myself again”*.

This ongoing communication provided a supportive environment for the participants to express their concerns, emotions, and aspirations. It enabled the OTs to tailor interventions and support mechanisms according to individual needs, making the rehabilitation process more effective.

### 3.3. Theoretical illuminations and reflections

Researchers have found that meaningful everyday activities can be powerful positive influences on health, wellbeing [59], and personal recovery [32]. However, for this target group in this context, the “everyday doing” does not come naturally—rather it has to be facilitated through conscious methods, participatory approaches, and staff collaboration.

#### 3.3.1. How the stories were told

The narratives and the identified storylines shed knowledge on **what** can be achieved in a relatively short time period, **how** it can be achieved, and moreover, **how** it is spoken about and interpreted in interactions through shared stories, experiences, and identified elements. Their stories are characterized by being straight to the point and concrete, identifying what has been helpful and putting into words how important elements worked.

In this study, all seven participants indicated that their support, offered to them during the ELR period, guided them to discover and develop through enrichening doing, being, and belonging, and furthermore, that it played a major role in their success of their everyday life rehabilitation and personal recovery process. Through the stories shared, we can also notice how this directly influenced the participants' personal recovery pathway, as they mentioned identity development, self-worth, hope, connectedness, etc.

Notably, the participants' stories contained several concepts and language from the ELR period, including motivational strategies and recovery components, indicating that they talked and reflected on this during their process and now re-used some of this wording in their own stories, integrated into their own understanding when they recalled how support and progress occurred. Concepts such as hope, inner motivation, possibilities, self-determination, values, meaning, identity, mattering, connectedness, and empowerment all relate to the recovery paradigm.

Through exploring the residents' rehabilitation stories and how they are narrated, the study offers insights into how the participants depict the significance and sense-making of their newfound engagement in everyday life activities, and how they attribute value in the support provided along their recovery processes. The narratives give voice and depth to their experiences and the major changes that took place over a short period of time. What they particularly emphasize regarding various aspects of the methods and support they received is completely in line with a person-centered approach in its true sense, which takes into account the unique and changing needs, as perceived and told by the person, the comprehensive perspectives, and the life circumstances of each individual [60],[61]. By adopting a person-centered approach, the ELR intervention aims to promote the participant agency and increase their engagement in their own recovery process and everyday life [62].

#### 3.3.2. Theoretical elaboration explicating how recovery through enrichening and meaningful activity took place

A theoretical perspective of doing-being-becoming-belonging, often used within occupational therapy contexts [42],[63], apparently relates closely to the recovery process. Proposing a “do-be-do-be-do-circular process” applied to the participants' stories could help in understanding how the doing of enrichening activity (input, meaning, growth, pride, emotions) influences one's being (mind, positive identity, self-worth, hope), which, in turn, influences the sense of belonging (social and societal connectedness) and becoming (developing skills, competences, roles, future prospects, empowerment/personal agency) in ongoing, inter-circular processes. Thus, how a negative spiral—often seen in supported housing context—can be turned into a positive spiral of hope, other impressions, worth, mattering, will, identity, personal agency, etc., even in persons who had previously reached a state of mind and level of sedentariness where they could not, by themselves, instigate change or describe what mattered to them.

Beside the rich stories of how doing activities influenced their personal recovery, the participants' stories, recalling their personalized rehabilitation processes, illustrated how support and signals of hope awakened inner motivation for personal progress, how weekly meetings supported participants' self-determination, identity, and exploration in activities, and how a transparent process and validating communication enabled their personal progress.

A recovery paradigm was applied in the ELR program and transformed to the personal processes; moreover, it was also applied as an overall theoretical framework in our narrative analysis to contextualize the findings and provide a theoretical lens to interpret the data, thus offering a broader perspective on the participants' descriptions of their experiences with ELR. The study findings suggest that meaningful activity can play a vital role in facilitating personal recovery. The participants' narratives underscore the crucial role of engaging in everyday life activities to foster hope, motivation, self-value, connectedness, mattering, and a sense of purpose in life. Participating in activities that they had chosen enabled participants to overcome the challenges they faced and to better manage their emotions while feeling valued, confident, and connected. The importance of rediscovering one's identity and cultivating a positive self-image through meaningful activity was emphasized by the participants. This aligns with existing discussions and research on the personal recovery paradigm within the context of supported accommodations, emphasizing the role of meaningful activities in facilitating the personal recovery of individuals with an SMI [64],[65].

## 4. Discussion and concluding remarks

The findings of this study indicated that the ELR focus on personally meaningful and enriching activities, achievable goals, and personalized support, can be highly valuable in facilitating personal recovery in individuals with SMI. By considering each individual's perspectives and life circumstances, the ELR intervention provided a tailored approach to rehabilitation that was perceived as relevant and meaningful to the participants. This approach allowed them to rediscover their sense of identity, motivation, hope, confidence, connectedness, sense of purpose, and agency, which are essential components of personal recovery [31].

In addition to the benefits of engaging in meaningful activity, our findings underscore the benefit of spending time outdoors and in nature for the participants' recovery process. The participants frequently mentioned how activities such as watching and sensing beautiful views, nature walks, shoveling snow, gardening, and skiing provided them with a sense of purpose, balance, calm, and connection with themselves and to the world around them. Our findings are consistent with studies which demonstrated that spending time in green and blue spaces [66],[67] has numerous mental health benefits, including an improved mood, reduced stress levels, and increased feelings of well-being.

The participants' stories highlighted the importance of the support they received to enable them to express their will, make choices, value, and engage in personally meaningful activities. Allowing the participants to make choices about their rehabilitation process and everyday life issues positively impacted their motivation, confidence, and commitment. This finding aligns with previous research which emphasized the importance of offering individuals the chance to make choices during their recovery process. When individuals feel empowered to make decisions, they are more likely to invest in their own recovery process and work towards their goals with greater dedication [33],[68],[69]. Therefore, rehabilitation programs that prioritize individual choices and involvement tend to be more effective in promoting positive outcomes for the participants [70].

All the participants' narratives emphasized the importance of achieving the goals set during rehabilitation. These findings are consistent with previous research that highlighted the importance of goal-setting in promoting successful outcomes in mental health interventions [71]. Self-chosen, clear, and achievable goals tailored to the individual's needs and abilities can help maintain motivation and keep the participants focused on the recovery process [35],[36].

### 4.1. Concluding remarks

These findings enhanced our comprehension of how engaging in meaningful activities can aid in the rehabilitation and recovery process of individuals with an SMI. Through the narrative analysis, we found that the participants created meaning through their engagement in everyday life activities in various ways, such as rediscovering personal identity and regaining self-confidence, re-awakening their motivation and lust for life, engaging in meaningful social roles, and improving social and physical skills. Some mentioned that it helped them manage their emotions and feel valued.

The participants also expressed how the opportunity to make choices about their rehabilitation process had a positive impact on their motivation and commitment. Spending time in social and/or outdoor spaces was also highlighted as a significant contributor to their sense of purpose, accomplishment, and connection to the world around them. The participants found meaning in engaging in activities that were self-chosen and tailored to their interests, needs, and abilities. Additionally, they underlined the importance of getting support during the process of exploration and change.

Overall, the narratives illustrated how engaging in meaningful activities within a person-centered, motivating, communicative, and supportive context could promote personal recovery among individuals with SMIs living in supported housing. By fostering a sense of value, meaning, autonomy, and social connectedness, programs such as the ELR have the potential to empower individuals to lead fulfilling lives despite the challenges they may face.

The study underscores the need for interventions that prioritize meaningful activities and are sensitive to the complexity of the personal recovery process, particularly within the unique context of supported housing facilities. Approaches that prioritize meaningful activities have shown promise in supporting personal recovery. Future research should continue to explore how these interventions can benefit individuals in these settings, with a focus on identifying the most effective strategies to promote personal recovery, a quality of life, and reducing the stigma associated with an SMI.

As persons with SMIs are given more opportunities to participate in meaningful everyday life activities, they may gain valuable tools and resources that help them discover their own true potential, shift a negative spiral towards a positive, develop a sense of purpose, and take ownership of their recovery journey, which can contribute to a fulfilling and meaningful life.

### 4.2. Methodological considerations

The material includes thick descriptions from a representative sample, which makes the findings transferable to a similar context. However, the results of this narrative inquiry study, which included seven participants of the ELR rehabilitation intervention, cannot be transferred to all people with SMIs living in supported accommodations. Nevertheless, it provides subjective and complex insights and directions for future studies that focus on intervention approaches to gain a deeper understanding of the activity-based and personal recovery pathways in rehabilitative practice communities.

The stories have shown how important it is to give voice and offers of rehabilitation to marginalized, easy-to-ignore groups. Allowing participants to share their stories and perspectives may provide an opportunity to recognize and appreciate their expertise as service users.

The analysis was enhanced through continuous comparisons and negotiations of the findings by the authors, using triangulation to accurately reflect the residents' experiences. In-depth subjective narratives and theoretical illumination contextualized the findings in the prevailing living conditions. This contextualization increases the possibility of relating the results to how other marginalized and disfavored residents with SMIs perceive their opportunities for transformation in everyday life.

As OTs and intervention-researchers in public health, aligning with health equity as well as narrative meaning-making, both authors' pre-understanding of the topic influenced the interactions and interpretations of the data collected from conversations with informants, and during the analysis phase [72]. Regarding reflexivity [72], throughout, we aimed to remain transparent, open-minded and critically reflective to our own perspectives' influence on the interpretations.

The predominantly positive stories shared by the participants can be attributed partly from the fact that six out of seven participants achieved their goals and the seventh participant came close to reaching her goal within the six-month rehabilitation phase. This period was described as an unexpectedly positive and transformative experience in their lives. From a critical perspective, this might partly relate to the fact that they were probably grateful to be given the chance to participate in the intervention. Furthermore, the positive stories could have been influenced by the role played by the interviewers, who presented ourselves as OTs that pursued research, which could possibly be associated with the guidance they received from their OTs during their ongoing change. It is also important to point out that the narrative approach and the type of conversational interviews that rendered the data material, and then formed the basis for analysis, included a dynamic interaction between the participants and the interviewers/analysts [72].

## Use of AI tools declaration

The authors declare they have not used Artificial Intelligence (AI) tools in the creation of this article.
